# Future research tendencies for solar energy management using a bibliometric analysis, 2000–2019

**DOI:** 10.1016/j.heliyon.2020.e04452

**Published:** 2020-07-22

**Authors:** Thamyres Machado David, Paloma Maria Silva Rocha Rizol, Marcela Aparecida Guerreiro Machado, Gilberto Paschoal Buccieri

**Affiliations:** a Department of Production Engineering, UNESP - Universidade Estadual Paulista, Guaratingueta, SP, Brazil; b Department of Electrical Engineering, UNESP - Universidade Estadual Paulista, Guaratingueta, SP, Brazil; c Department of Energy, UNESP - Universidade Estadual Paulista, Guaratingueta, SP, Brazil

**Keywords:** Energy, Green engineering, Sustainable development, Energy management, Solar energy, Photovoltaic solar energy, Bibliometry

## Abstract

Using the Scopus database between the years of 2000 and 2019, a bibliometric study was done to analyze the scientific publications in the area of photovoltaic solar energy management. From the preliminary analysis of future research tendencies, ten possibilities of study topics were developed; and due to that it was possible to assume that even though many studies of technological development are found, some insights can still be approached in a way that the practical implementation of solar systems photovoltaic is better used. This data was validated by the analysis performed with the Scimat scientific mapping software under a longitudinal structure, verifying the future tendencies researches mentioned previously.

## 1 Introduction

The production of energy can cause environmental impact due to some pertinent factors such as the use of fossil fuels, and deforestation. With the gradual increase in energy consumption in the world, the use of renewable energy has grown with the use of natural resources that will not have a negative effect in the environment and by the generation of clean energy like the sun and the wind.

The increase in the energy demand and how we are going to supply it is one of the world's major issues and challenges [1]. Thus, the set of resources and changes in non-fuel sources of the energy system (i.e., wind, solar) become more prominent with the progress of the energy transition. Those fuels are different from fossil in two fundamental aspects: they are abundant, not scarce, but its instantaneous availability is limited, instead of being dispatched on demand [2].

The source of PhotoVoltaic (PV) solar energy is obtained through photovoltaic panels; being an abundant and sustainable source of energy, that is highly promoted for its environmental privileges because it does not emit harmful gases, such as carbon dioxides, contributing to the slowing of global warming and to meet global energy demand in a sustainable way [3]. The research, development and continuous improvement of this solar technology is essential in order to increase the energy efficiency for beneficial use in all consumer segments [4, 5].

Due to the importance of the subject previously mentioned, solar PV energy management has been an important research topic since the 1970s. Between 1990 and 2000, the few articles found were focused on two themes: solar energy for heating [6, 7, 8, 9], and studies and development of solar energy in advanced countries [10, 11, 12, 13, 14] aimed at the application of this type of generation. During this period, there were no significant publications regarding energy management because of the recentness of the subject; Significant publications began in early the 2000s [15, 16, 17, 18].

In more recent studies on photovoltaic solar energy management, authors addressed the environmental performance of photovoltaic systems with life cycle assessment studies and efficiency improvements achieved in the conversion of solar cell devices [19, 20, 21]. Guidelines for comparison of solar energy forecasts have been identified as well as the prediction of certain factors that influence PV generation in other recent studies [22, 23].

This article intends to present a bibliometric and bibliographic analysis of publications on photovoltaic solar energy management in the period of 2000–2018. The term "bibliometrics" was first used by Pritchard [24] in his article “statistical bibliography or bibliometrics?”. According to the author, the bibliometric method is the application of mathematical and statistical methods to books and other means of communication. For Bellis [25], the bibliometric sums up a set of methods to quantitatively analyze the scientific literature and technology.

Table 1, which includes four bibliometric articles found in the scientific literature that somehow approach solar PV energy, was developed aiming to explain the inexistence of publications with bibliometric features related to the solar PV energy management term. It presents the author's name, the journal where it was published, as well as the article's title and goal. Although bibliometric studies have already been published in solar and all it subareas, there are researchers presenting the same level of detail and analysis made in this study; offering any kind of broad approach, for example, the extensive trend approach of future studies related to the subject, were not found in the scientific literature, being this a great differential of the present article.

**Table 1:** Bibliometric articles found in the scientific literature that approaches solar PV energy.

Author(s)	Name of publication journal	Article Title	Object
Du, Li, Brown, Peng and Shuai [26]	Renewable Energy	A bibliographic analysis of recent solar energy literatures: The expansion and evolution of a research field	The main objective of this paper is investigate the characteristics of the solar energy literature from 1992 to 2011 and its implication using bibliometric techniques taking into account solar energy's expanding and shifting focus.
Paulo and Porto [27]	Energy Policy	Solar energy technologies and open innovation: A study based on bibliometric and social network analysis	The study aims to identify the development of solar energy technologies through open innovation.
Naves, Barreneche, Fernández, Cabeza, Haddad and Boer [28]	Solar Energy	Life cycle costing as a bottom line for the life cycle sustainability assessment in the solar energy sector: A review	The study aims to prove that the Solar Energy sector, that deals with one of the most important renewable energy resources, has been a close relationship with Life cycle costing.
Calderón, Barreneche, Hernández-Valle, Galindo, Segarra and Fernández [29]	Solar Energy	Where is Thermal Energy Storage (TES) research going? – A bibliometric analysis	The main objective of this paper is to provide an overview of the history of solar thermal energy storage research and development, by using bibliometric methods.

Before going any further, and based on the scientific gap, we have established the following research question that oriented the development of this article: how does the bibliometric and bibliographic analysis regarding solar PV energy management can provide a future research tendencies approach?

This article presents a comprehensive assessment of the solar PV energy management field; beginning with a survey of over 3,260 published articles and filtering this group to more influential articles and researchers. From these results, information about current research interests and potential directions for future researches are obtained. As a way of determining the point of view approached in this study, the technical-engineering aspect of the photovoltaic sector is considered [30, 31, 32]. This paper is divided into four sections, including the introduction. The following section presents the method that was used as well as the steps in the article. The third section presents the main results and discussions, and in the fourth section, the conclusion.

## 2 Methodology

The first step of the bibliometric study was the search in the Scopus database to obtain a set of data for all published articles related to solar PV energy management. Scopus is a field of bibliographic research, founded in 2004 by Elsevier; that indexes citations for scientific publications. The analysis interval was between the years 2000 and 2019.

To analyze the data of these documents, their corresponding metadata was imported into Microsoft Excel 2010. Scopus allows us to import files directly from the database; which assist us to identify which articles are exclusively related to the solar energy management limited to photovoltaic (PV) type, the titles and abstracts of all the articles were analyzed. Those which were not in the scope of this research were eliminated after checking the text of the article itself. The keywords used for data collection included "solar”, “energy", "management" and "photovoltaic". We used one combination of these keywords with Boolean operators to combine the search terms as follows:

(i) Photovoltaic solar energy management:

(“solar” and “energy” and “management” and “photovoltaic”)

In this study, we only focus on papers published after the 2000s, due to the fact that there was less data regarding photovoltaic solar energy management before that year. To shed the light on photovoltaic solar energy management trends and contributions, bibliometric and qualitative analysis are conducted in this research.

For the analysis of future research tendencies, a bibliographic analysis was made with also the use of the Scimat software; which is a tool developed to perform science mapping analysis under a longitudinal structure, as a way of validating the data and for the analysis of the co-author's countries and keywords co-occurrence, VoSviewer software was used following the same methodological basis used by Martinho [33]. The software VoSviewer can be applied to develop maps based on network information and for visualizing and analyzing maps. The maps created include items (terms, publications, or authors) and, between the items, there are links (the strength of each link is indicated by a value). In this context, for example, co-occurrences are links among terms and the strength of the links, in these cases (co-occurrences), shows the number of publications in which the terms appear together. In the network map, the software represents each item via a label and a circle, their size is dependent upon the item weight and the lines between the items are relative to the links. On the other hand, the distance between the items presents their relatedness.

## 3 Results and discussions

### 3.1 Co-authors evolution: countries

**Figure 1:**
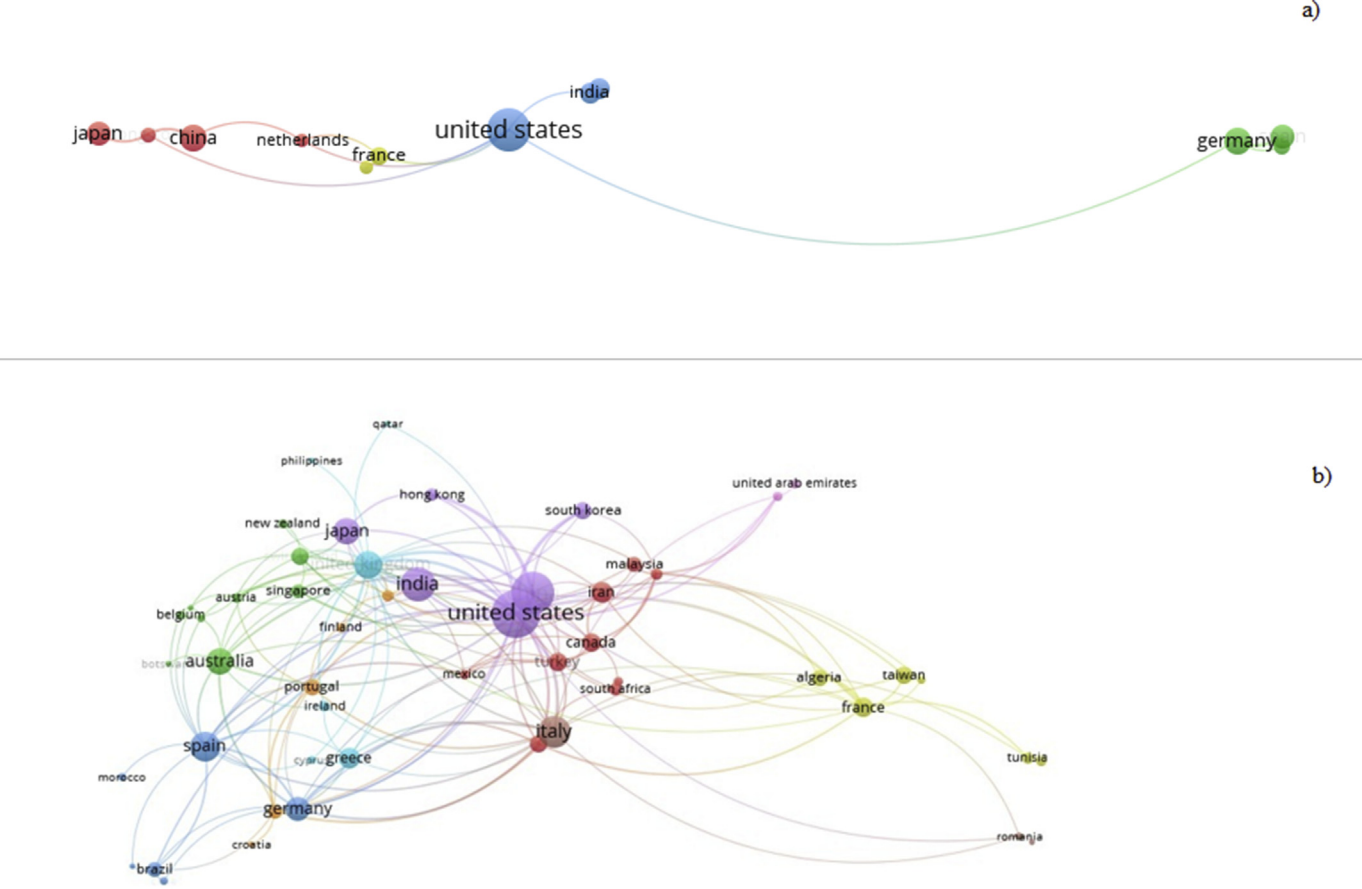
Co-authors countries: a) 2000 to 2010 and b) 2011 to 2018.

The VoSviewer software was used to detect the co-authors' countries and to analyze the interactions between them. In the first analysis, performed during the period from 2000 to 2010 (Figure 1a) only 10 countries, 3 clusters and 11 links were identified. In the second analysis, performed during the period from 2011 to 2018 (Figure 1b), in which there was a significant increase in the relation in comparison to the previous period, 59 countries, 9 clusters and 183 links were returned. Each node represents a country, and every publication creates a link between the co-authors. With the records, we consider each country as a node and all collaboration between two authors as an advantage.

There are three major countries with the greatest impact in this area which are the United States, India and China, respectively. Node size represents the country's comprehensive impact. Thus, the bigger the node is, the higher collaborative capability and impact the country is. Within the countries that have most related with the author, it is noteworthy that the three countries who are highlighted were countries leaders in the solar energy segment, proving the investment in research in this area is a way to develop this kind of power source.

### 3.2 Keywords co-occurrence analysis

**Figure 2:**
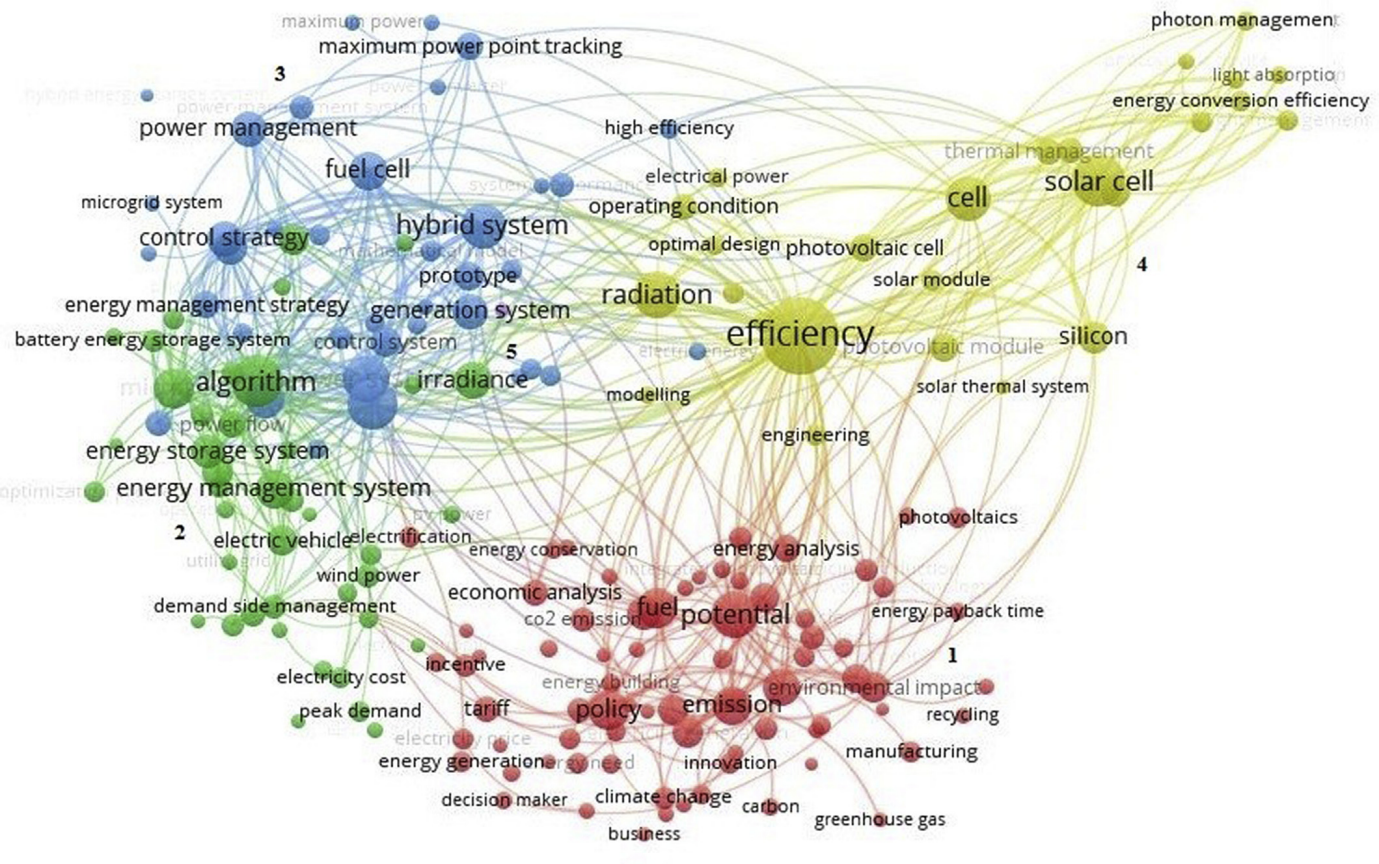
Keywords co-occurrence.

Based on keyword analysis, we get some basic knowledge about the keyword distribution in solar PV energy management, which establishes a solid foundation of keyword co-occurrence frequency analysis. We produced a network of title and abstract co-occurrences (Figure 2) based on solar PV energy management articles using Scopus database data and software VOSviewer [34].

To extract keywords from the titles and abstracts, it was used by the text mining function of VOSviewer. This function creates a co-occurrence network of keywords (adjectives and nouns) and displays it on a two-dimensional map. Two keywords are said to co-occur if they both occur in the same title/abstract and the keywords with a higher rate of co-occurrence tend to be found closer to each other.

In this study, to generate the co-occurrence map, the following settings were used in VOSviewer: we used binary counting, a keyword had to occur at least eight times and the number of clusters was determined based on interpretability reasons. Keywords not relevant to our analysis were excluded manually and words that structure abstracts like "case studies", "theory", and "review" were removed.

A total of 194 keywords were extracted that occurred eight or more times (excluded the keywords that did not apply to the study). As can be seen in Figure 2, 5 clusters were created and cluster 1, with 76 keywords, basically addresses managerial issues of PV solar and environmental corroborating with the expectations of the authors. Cluster 2 (47 keywords) addresses more technical issues and transmission grid. Cluster 3 (41 keywords) addresses the maximum energy power and control systems, cluster 4 (29 keywords) on efficiency and solar cells and, lastly, cluster 5 (1 keyword), that cannot be observed in Figure 2, addresses only available energy.

By the analysis of the network, where we get 201 nodes and 5427 connections between them, it can be observed that the network of co-occurrence of keywords clearly showed the main aspects addressed in the area and also provides an approximate view for future research.

### 3.3 Future research tendencies

**Figure 3:**
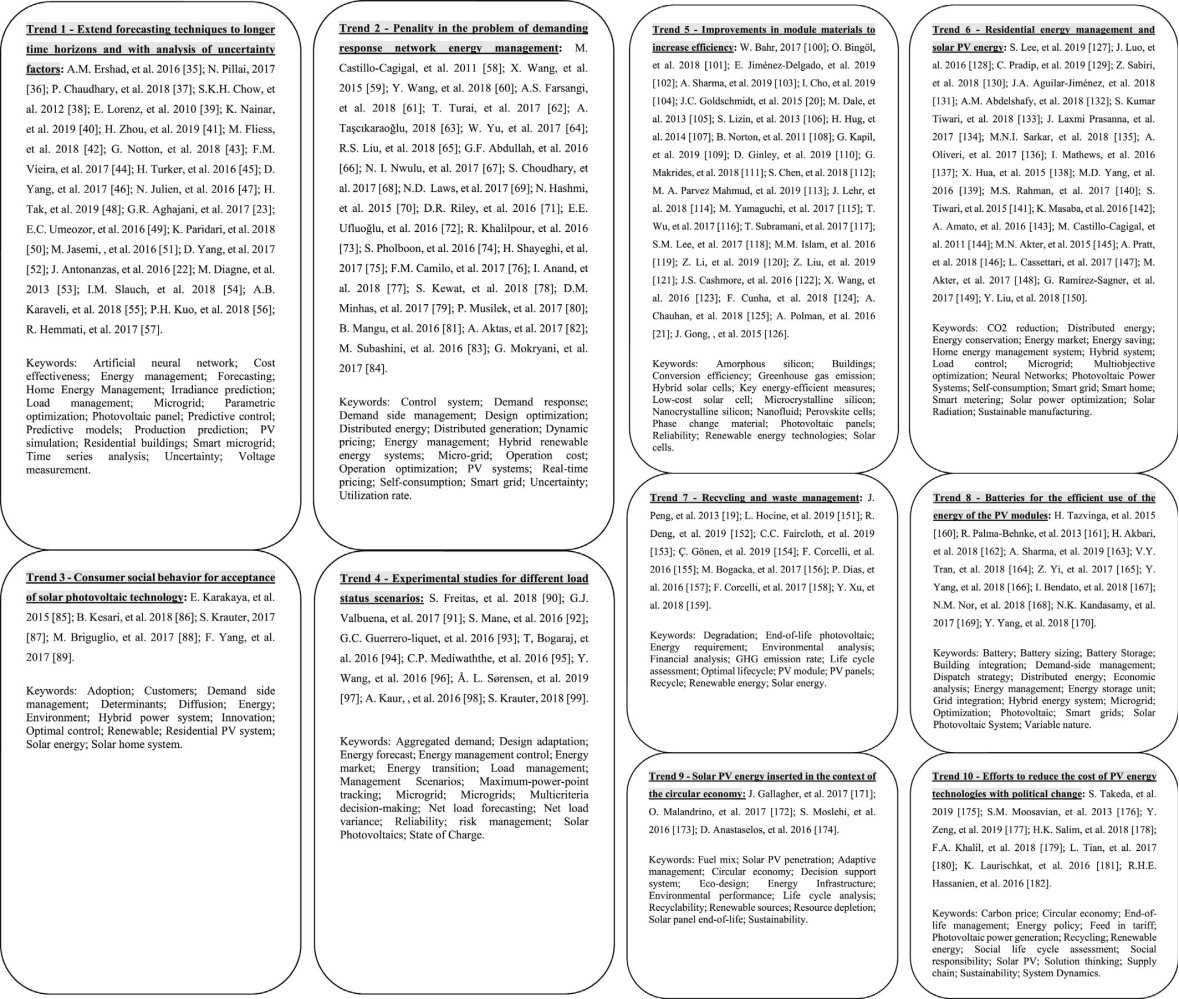
Articles with future research tendencies: qualitative analysis of articles published between 2010 and 2019 (153 articles mappeds). (Trend 1) Extend forecasting techniques to longer time horizons and with analysis of uncertainty factors; (Trend 2) Penality in the problem of demanding response network energy management; (Trend 3) Consumer social behavior for acceptance of solar photovoltaic technology; (Trend 4) Experimental studies for different load status scenarios; (Trend 5) Improvements in module materials to increase efficiency; (Trend 6) Residential energy management and solar PV energy; (Trend 7) Recycling and waste management; (Trend 8) Batteries for the efficient use of the energy of the PV modules; (Trend 9) Solar PV energy inserted in the context of the circular economy; (Trend 10) Efforts to reduce the cost of PV energy technologies with political change.

To evaluate future research tendencies, the articles which were published between 2010 and 2019 were analyzed respecting the scientific literature gaps which they propose. Of the 3260 articles on solar PV energy management that were published between 2010 and 2019, 235 were not accessible, 955 did not apply to research and 1917 did not include proposals for further research (Figure 3) [35, 36, 37, 38, 39, 40, 41, 42, 43, 44, 45, 46, 47, 48, 49, 50, 51, 52, 53, 54, 55, 56, 57, 58, 59, 60, 61, 62, 63, 64, 65, 66, 67, 68, 69, 70, 71, 72, 73, 74, 75, 76, 77, 78, 79, 80, 81, 82, 83, 84, 85, 86, 87, 88, 89, 90, 91, 92, 93, 94, 95, 96, 97, 98, 99, 100, 101, 102, 103, 104, 105, 106, 107, 108, 109, 110, 111, 112, 113, 114, 115, 116, 117, 118, 119, 120, 121, 122, 123, 124, 125, 126, 127, 128, 129, 130, 131, 132, 133, 134, 135, 136, 137, 138, 139, 140, 141, 142, 143, 144, 145, 146, 147, 148, 149, 150, 151, 152, 153, 154, 155, 156, 157, 158, 159, 160, 161, 162, 163, 164, 165, 166, 167, 168, 169, 170, 171, 172, 173, 174, 175, 176, 177, 178, 179, 180, 181, 182].

To precisely clarify the subject areas of the identified trend groups, the keywords of each article were selected and those that had an incidence of two or more in the articles of the group were selected (Figure 3). It can be noticed that nine keywords: Photovoltaic panel; Demand side management; Energy management; Distributed energy; Hybrid energy system; Renewable energy; Smart grid; Microgrid; Sustainability are the most used by the authors in comparison to all groups of words.

Therefore, only the 153 remaining articles mapped in Figure 3 were analyzed. We divided the gaps according to the scientific literature of these 153 articles into 10 topics (illustrated as large rounded boxes), as seen in Figure 3:

(Trend 1) Extend forecasting techniques to longer time horizons and with analysis of uncertainty factors. Considering the differences between operational and laboratory forecasts; a study to complement the stability of the network at night.

(Trend 2) Penality in the problem of demanding response network energy management; Demand management should consider the response mechanism of the electricity load.

(Trend 3) Consumer social behavior for acceptance of solar photovoltaic technology.

(Trend 4) Experimental studies for different load status scenarios; system to control disconnections of load groups; adequate management strategy to mitigate the variation of the uncontrollable net load.

(Trend 5) Improvements in module materials to increase efficiency; find replacement parts or processes; optimize thermophysical properties and the spectral reflection of the lower layer materials for maximum heat dissipation and higher energy yield; application of biophotovoltaic; understand mechanisms of module degradation in different climates and reduce the consumption of precious metals.

(Trend 6) Residential energy management and solar PV energy: Balance of energy produced by PV panels and consumption by users' devices; communication with other consumers and providers of distribution network services; consumer adoption of home automation products; hybrid energy system for domestic application; evaluation of rigorous techniques must be carried out before the integration of solar PV energy in the distribution networks. Internet of things: designing the efficient communication scheme between several smart homes to achieve energy savings.

(Trend 7) Recycling and waste management: recycling facilitated by the appropriate design of the photovoltaic module to facilitate the separation of components; compare the cost of charges caused by pollutants to the cost of reducing that pollutant; study for the extended life cycle of the panels; policies and regulations to encourage recycling and the safe disposal of waste.

(Trend 8) Batteries for the efficient use of the energy of the PV modules: Dimensioning of the battery considering hybrid criteria; definition of standards for storage of electricity in the PV system; PV system analyzed with the Usage Time Tariff.

(Trend 9) Solar PV energy inserted in the context of the circular economy.

(Trend 10) Efforts to reduce the cost of PV energy technologies with political change; firmly incorporate socio-economic factors and tax incentives; evaluation of PV projects at the macro level; integration of the PV system to other renewable energy sources and flexible retail financing terms.

In future mapped research tendencies, there is a wide variety of issues from the identified gaps. Although some mapped trends refer to economic factors, this is not addressed in a primary way compared to other research gaps. Seetharaman et al. realized that economic barriers do not directly affect the usage and improvement of renewable energy, but they are co-related to social, technological and regular barriers, affecting the deployment of renewable energy not directly [1].

The Scimat software was used to validate the results obtained with the analysis of the research gaps. Such software is a tool developed to perform science mapping analysis under a longitudinal structure that provides different modules. Different normalization and similarity measures can be used on the data and several clustering algorithms can reduce the data. In the visual module, three representations (strategic diagrams, cluster networks and areas of evolution) are used together [183].

The software shows, through the strategic diagram, in which category the analyzed term is found. Each quadrant of the x and y axes has its own peculiarity of classification. The first quadrant is classified as motor, that is, they are themes developed and important to structure a research field. The consolidated themes are found in the second quadrant, that is, internally well-developed themes, but isolated from other themes and are of marginal importance for the development of the research field. The third quadrant presents the classification that varies among the others, because in this quadrant the obsolete (old themes) or incipient (starting in the research field) are considered. Its actual classification will depend on the evolutionary analysis of the term, that is, if the term is tending to go out of the diagram it is considered obsolete, on the other hand, if it tends to go to the fourth quadrant, it is considered an incipient term. The fourth and last quadrant is classified as transversal, that is, they are important for a research field, but not enough developed [184].

**Figure 4:**
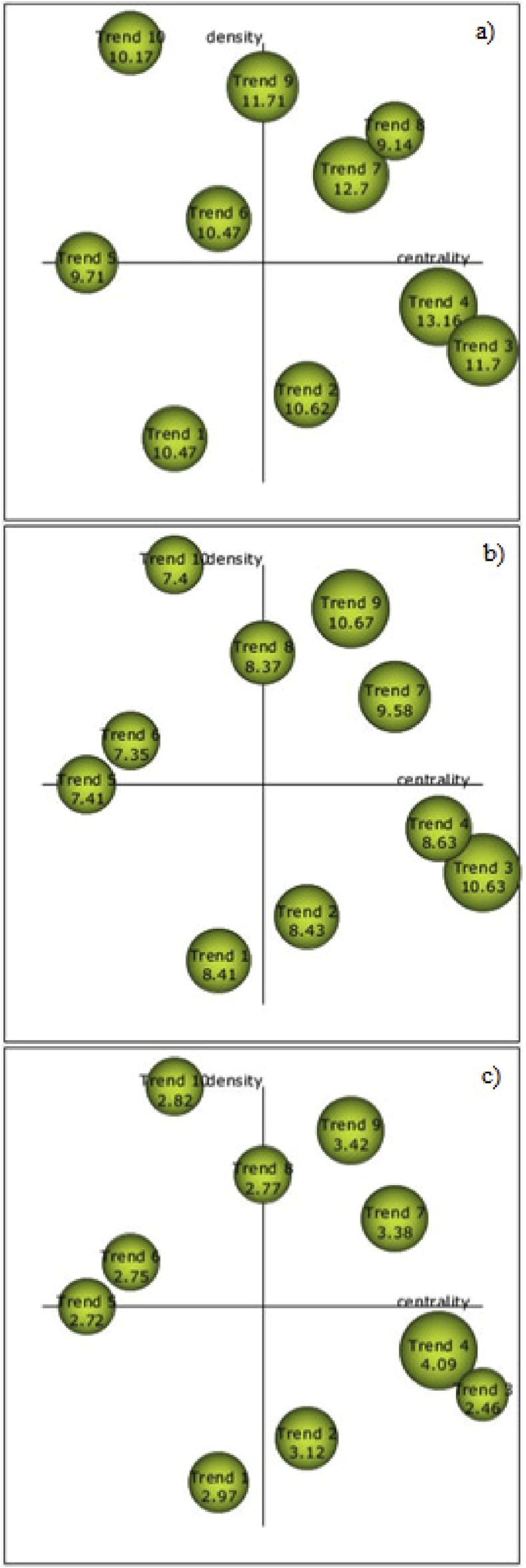
Strategic diagram of the periods 2017 (a), 2018 (b) and 2019 (c).

For the analysis, 648 keywords were structured and divided into the 10 trends identified in the articles. The strategic diagrams were based on the principles of centrality (analyzes the strength of the relationship of external links with other themes, shows the importance of developing a theme in a research field) and density (which assesses the internal strength of the network, analyzing the internal links among keywords grouped around a specific topic) [185]. The period of analysis was from 2017 to 2019 to determine the evolution of the groups and it was divided using the manager period of the bibliometric software. With the results shown in the diagram in Figure 4, follow the comments about each:

Trend 1: the topic is in the third quadrant in the period of 2017, has being considered incipient and gradually moves to the fourth quadrant in 2018, when it becomes a transversal topic with other research subjects. Even having a lower relevance in 2019 (representative sphere size based on a smaller research than in 2017 and 2018), it is considered a base trend of three important trends (2, 4 and 8).

Trend 2, 3 and 4: the topics are in the fourth quadrant in all the periods searched, being a transversal topics with other research subjects. Trends 3 and 4 are considered to be the last representative thematic areas.

Trend 5 – the topic emerged as an isolated theme and incipient in all periods searched (second and third quadrant), that is, the topic has relevance in an isolated way in the context of the analyzed articles and being considered emergent in the development of studies on the theme. However, there is a decrease in intensity in 2019 even though it is the most representative thematic area in the conceptual map of evolution according to the h-index.

Trend 6: the topic developed as an isolated theme in all periods searched (second quadrant), that is, the topic has relevance in an isolated way in the context of the analyzed articles and it is considered the second most representative thematic area on the map.

Trend 7: the topic emerged as a motor theme in all periods looked for (first quadrant), that is, the topic is presented in most studies in the area.

Trend 8: the topic is developed as a motor theme in 2017 (first quadrant) and it is an isolated theme in 2018 and 2019 (second quadrant). In this specific trend, less development can be seen in the following periods, being considered an important problem. Considering that the theme of the trend involves batteries for the efficient use of the energy of the PV modules, studies point out the difficulty of funding this equipment without political support [186,187].

Trend 9: the topic emerged as a motor and isolated theme in 2017 (first and second quadrant) and a motor theme in 2018 and 2019 (first quadrant).

Trend 10: the topic emerged as an isolated theme in all periods searched (second quadrant), that is, the term has relevance in an isolated way in the context of the analyzed articles. But, although the intensity tends to decrease in 2019, the topic tends to become a motor theme and it is considered the third most representative thematic area on the map.

## 4 Conclusion

This article shows some research possibilities of the database about solar PV energy management through bibliometric techniques, which can be obtained with the purpose of describing, knowing or explaining phenomena. It is noted that, as far as we know, a study on the researched topic has not been carried out yet and that the highest interest concentration is inserting it in the context of optimizing processes of solar energy generation, as well as the elements prediction that contributes to this generation. All in all, the field of solar energy management is growing and maturing, it has been found more than 1150 articles in the filtration. However, when we analyze the articles by the technical aspect the number of publications decreases abruptly. It is proved that there is still a significant room for development given the small number of relevant articles when related to energy management.

In the keywords, the results show that the degree of knowledge diversity is high, as demonstrated by the increase of distinct keywords. For analysis, we produced a network of title and abstract co-occurrences that clearly showed the main aspects addressed in the and also provided an approximate view for future researches.

In relation to authors and institutions, the VoSviewer software was used to detect the countries of the co-authors and to analyze the interactions between them. The co-author network was highly fragmented and the clustering coefficient is not very high, indicating that only a few authors tend to form closely clustered agglomerates.

In relation to future research, ten main tendencies were identified: (1) Forecasting techniques; (2) Demand management; (3) Consumer social behavior for acceptance of solar photovoltaic technology; (4) Load status studies; (5) Module materials to increase efficiency; and understand mechanisms of module degradation in different climates; (6) Residential energy management and solar PV energy (7) Recycling and waste management; (8) Batteries for the efficient use of energy from the PV modules; (9) solar PV energy inserted in the context of the circular economy; and (10) Efforts to reduce the cost of PV energy technologies through political changing.

The ten trends identified bring study opportunities that address, for example: multi-objective optimization to maximize forecasting techniques for factors such as solar radiation, wind speed, shading, temperature with uncertainty analysis and the use of smart meters; control of energy flow using a bidirectional meter consisted by ultra-capacitors and inclusion of fines, such as fines for barriers to response demand; increase in experimental studies to improve module materials or even discover of new components; giving emphasis on waste management of the modules, relating to this fact being considered of secondary order due to the fact that the PV module has a long life cycle; emphasis on applications to make batteries competitive in the market and dissemination of the theme in the context of the circular economy to gain more social reach.

Content analysis of the research gaps was verified in the Scimat software. The terms shown in Figure 4 are in the quadrants considered relevant in the literature, since they are consolidated, motor, transversal and incipient themes. The terms found in the third quadrant are not characterized as obsolete themes, due to their tendency to go to the fourth quadrant, becoming incipient themes. Of the 10 research trends identified, nine were or tended to move towards motor themes or transversal themes, considered as themes that favor the development and consolidation of a field of knowledge. When analyzing these themes and their evolution, the research finds that the development of PV energy management will mainly support technical aspects as identified in 5 trends (trends 1, 2, 4, 5, 6 and 8) and socioeconomic aspects as identified in 4 trends (trends 3, 7, 8 and 9). In general, the interests of researchers who explore the research theme of the article should be focused on aspects related to recycling and waste management, batteries for the efficient use of energy from PV modules and solar PV energy inserted in the context of the circular economy because these are the trends that appear most in the motor themes of the diagrams.

It was possible to realize that, although many studies on the current theme are found, some ideas can still be approached in a way that the practical implementation of photovoltaic solar energy is disseminated. This article may assist researchers in finding ideas for future studies on solar PV energy management, identifying which topics cannot be left incomplete in their researches. This bibliometric analysis stimulates the revision and consolidation of already existing directions in this field of research and the exploration of new ones. As the main scientific contributions of this paper, we have the presentation of the solar PV energy management theme, still few explored as can be observed in the analysis and its growing importance to the scientific community in order to encourage further studies and research on the subject. This article can help researchers find ideas for future studies on photovoltaic solar energy, identifying which topics cannot be left unfinished, and policy governors on transversal, governance, social, legal or political issues for their studies.

## Declarations

### Author contribution statement

All authors listed have significantly contributed to the development and the writing of this article.

### Funding statement

This work was supported by the 10.13039/501100002322Coordenação de Aperfeiçoamento de Pessoal de Nível Superior [001].

### Competing interest statement

The authors declare no conflict of interest.

### Additional information

No additional information is available for this paper.
